# Training Multilayer Perceptron with Genetic Algorithms and Particle Swarm Optimization for Modeling Stock Price Index Prediction

**DOI:** 10.3390/e22111239

**Published:** 2020-10-31

**Authors:** Fatih Ecer, Sina Ardabili, Shahab S. Band, Amir Mosavi

**Affiliations:** 1 Department of Business Administration, Afyon Kocatepe University, Afyonkarahisar 03030, Turkey; fecer@aku.edu.tr; 2 Biosystem Engineering Department, University of Mohaghegh Ardabili, Ardabil 5619911367, Iran; s.ardabili@ieee.org; 3 Kando Kalman Faculty of Electrical Engineering, Obuda University, 1034 Budapest, Hungary; 4 Future Technology Research Center, College of Future, National Yunlin University of Science and Technology, 123 University Road, Section 3, Douliou, Yunlin 64002, Taiwan; shamshirbands@yuntech.edu.tw; 5 Faculty of Civil Engineering, Technische Universität Dresden, 01069 Dresden, Germany; 6 Institute of Research and Development, Duy Tan University, Da Nang 550000, Vietnam; 7 School of Economics and Business, Norwegian University of Life Sciences, 1430 As, Norway

**Keywords:** stock market, machine learning, multilayer perceptron, financial data, artificial intelligence, artificial neural networks, online trading, big data, social science data, evolutionary algorithms, optimization

## Abstract

Predicting stock market (SM) trends is an issue of great interest among researchers, investors and traders since the successful prediction of SMs’ direction may promise various benefits. Because of the fairly nonlinear nature of the historical data, accurate estimation of the SM direction is a rather challenging issue. The aim of this study is to present a novel machine learning (ML) model to forecast the movement of the Borsa Istanbul (BIST) 100 index. Modeling was performed by multilayer perceptron–genetic algorithms (MLP–GA) and multilayer perceptron–particle swarm optimization (MLP–PSO) in two scenarios considering Tanh (x) and the default Gaussian function as the output function. The historical financial time series data utilized in this research is from 1996 to 2020, consisting of nine technical indicators. Results are assessed using Root Mean Square Error (RMSE), Mean Absolute Percentage Error (MAPE) and correlation coefficient values to compare the accuracy and performance of the developed models. Based on the results, the involvement of the Tanh (x) as the output function, improved the accuracy of models compared with the default Gaussian function, significantly. MLP–PSO with population size 125, followed by MLP–GA with population size 50, provided higher accuracy for testing, reporting RMSE of 0.732583 and 0.733063, MAPE of 28.16%, 29.09% and correlation coefficient of 0.694 and 0.695, respectively. According to the results, using the hybrid ML method could successfully improve the prediction accuracy.

## 1. Introduction

Accurately predicting the stock market (SM) index direction has frequently been a topic of great interest for many researchers, economists, traders and financial analysts [1]. Nonetheless, the SM field is neither static nor predictable. In fact, SM trends are sensitive to both external and internal drivers. Thus, SM index movement estimation can be categorized under complex systems [2]. Stock price movement is often interpreted as the direction of stock price and used for prediction. Determining the future direction of stock price movement is of utmost importance to investors to evaluate the market risks. Predicting direction of stock price movement has been seen as a challenging and complex task to model [3]. Complex system is a framework to work how a system’s sub-categories interact with each other and how the whole system interacts and manages relationships with its environment. Whereas modeling such complex systems, challenges have encountered during constructing a reliable and effective technique and deciding its architecture [4].

Stock price movement forecasting is a compelling task because of the high volatility, anomaly and noisy signal in the SMs’ area. Over the past two decades, this topic has attracted the attention of researchers in different fields, particularly artificial intelligence [5]. Stock prices are nonlinear with regard to historical data and other technical and macroeconomic indicators [6]. Many researchers often preferred to use time-series analyses which is utilized to estimate future events according to historical data before the capabilities of neural networks were discovered. Autoregressive integrated moving average (ARIMA), autoregressive conditional heteroskedasticity (ARCH) model and Generalized autoregressive conditional heteroskedasticity (GARCH) model, support vector machine (SVM) are among the best-known models among the methodologies [7]. Moreover, regression analysis and artificial neural networks (ANNs) have frequently been used for forecasting and classification in order to cope with these nonlinear relationships [7,8,9,10]. Systems that utilize technical analysis, across expert systems, hybrid systems and various types of computational intelligence have as well been suggested [11,12,13]. Interests of researchers continue to increase for applying different types of artificial intelligence to forecast SM index direction. Due to the nonlinear structures of the problems, the prediction approaches are typically highly complex, meanly needing to develop efficient solution methods for such models. Technical analysts work with many data, technical tools and especially technical indicators to decide price trends and market trends on the basis of price and volume conversions in the market [14]. In light of existing literature, several technical indicators have been preferred as input data in the creating of forecasting methodologies to predict the direction of a SM index [15,16,17,18,19,20,21,22]. Cervelló Royo and Guijarro, 2019 employed four ML based prediction methods including gradient boosting machines (GBM), random forest (RF), generalized linear models (GLM) and deep learning (DL) for to address the estimation of market trends as a comparison analysis by accuracy rate (%) [23]. 

In several studies, technical indicators are used as input data. The trend was to identify novel methods that provide the highest accuracy for the classification methods in the index direction prediction. When we provide real-valued technical indicators as inputs to the models, forecasting techniques to be classified in accordance with the values of technical indicators are created [15,20]. When technical indicators are utilized, the prediction models consider each indicator as input data, regardless of the index being considered [7]. The studies in which the methods that deal with technical indicators as input data are used in the index direction prediction constitute the framework of this study. Estimating the BIST 100 index’s direction is a significant financial issue that has carefully monitored in financial markets around the world [24,25]. In this context, this research aims to predict stock price movement direction through an integrated multilayer perceptron methodology. More specifically, two novel models, i.e., multilayer perceptron–genetic algorithms (MLP–GA) and multilayer–particle swarm optimization (MLP–PSO) with Tanh (x) as the output function, have been proposed and applied for prediction compared with the default Gaussian function. Thus, it is intended to fill a gap in SM direction prediction literature. PSO has been employed by researchers for the prediction of stock market. Lahmiri, 2018 developed spectrum analysis and support vector regression integrated with PSO to estimate the stock price using time series data [26]. Pulido et al., 2014 employed PSO for the optimization of a hybrid ANN–Fuzzy model for the prediction of Mexican Stock Exchange [27]. Lahmiri, 2016 employed PSO for the optimization of the architecture of a feed forward neural network in the prediction of stock market [28].

The rest of this research is organized as follows: The next section reviews the relevant literature. Section 3 deals with the research methodology. The results are given in Section 4, while the findings are discussed in Section 5. The conclusions are presented in the final section.

## 2. Literature Review

In recent years, there have been a great deal of papers investigating the direction of the next day trends of SMs. Academicians and traders have made enormous efforts to forecast the next day trends of SM index for translating the predictions into profits (Kara et al., 2011). In this section, we focus the review of methods and technical indicators utilized for forecasting of direction movement of stock index. As shown in Table 1, an ANN model was used in some of the studies [18,25,29,30], whilst hybrid models were preferred in other studies [17,21,31,32,33] as displayed in Table 2. In Table 1. the notable algorithms are back-propagation neural network (BPNN), independent component analysis- BPNN (ICA–BPNN), Naive Bayes (NB), and k-nearest neighbors algorithm (*k*-NN). 

Table 1 summarizes several machine learning methods proposed for stock exchange index direction prediction. The most popular methods are RF, SVM and ANN, followed by *k*-NN and NB, with dissimilar accuracy outcomes. The state of the art shows a research gap in using hybrid models. 

Table 2 summarizes the notable machine learning models. Hybrid models of the SVM trained with simple evolutionary algorithms such as GA have been the most popular. The state of the art of hybrid models shows a research gap in using more sophisticated machine learning models trained with advanced soft computing techniques.

### Technical Indicators

As mentioned above, technical indicators have been useful and effective financial instruments for estimating direction of stock price index for years. Technical indicators used for SM direction prediction from past to present can be seen in Table 3.

In summary, MACD, %K, %D, RSI, %R, A/D, MOM, EMA, CCI, OSCP and SMA are the technical indicators that are frequently preferred by researchers. Furthermore, ANN and its extensions (MLP, PNN, etc.) are the most used methods. As far as the authors know, MLP–GA and MLP–PSO methodologies with and without Tanh (x) as the output function have not been proposed to forecast stock exchange movement prediction for any stock exchange in the literature. As a result, it is anticipated that this paper will constitute a significant contribution to the related field.

## 3. Materials and Methods

### 3.1. Data

As shown in Table 4, nine technical indicators for each trading day were utilized as input data. Plenty of investors and traders handle certain criteria for technical indicators. A great deal of technical indicators is available. As already mentioned above, technical indicators have often been considered as input variables in the construction of forecasting systems for estimating the trend of movement of SM index [24]. As a result, we determined nine technical indicators by previous studies and the opinion of area experts.

The input variables utilized in this work are technical indicators described in Table 4 and the direction of change in the daily Borsa Istanbul (BIST 100) SM index. The entire data contains the period from 28 March 1996 to 7 February 2020, providing a total of 5968 trading day observations. Furthermore, information about opening and closing price is available for each trading day. The number of entire data with decreasing direction is 2827 (47.36%), whereas the number of entire data with increasing direction is 3141 (52.64%). All the data were obtained from Matriks Information Delivery Services Inc. (https://www.matriksdata.com).

### 3.2. Methods

#### 3.2.1. Multilayer Perceptron (MLP)

**Figure 1:**
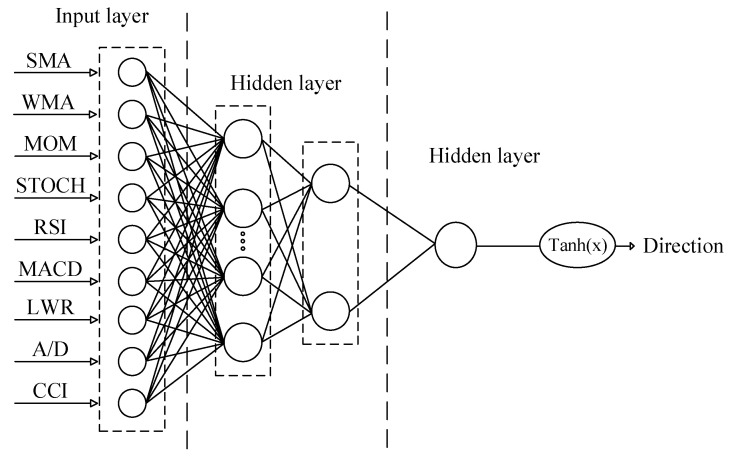
The architecture of MLP.

The architecture of ANN is based on connections of layers by nodes called neurons as well as the biological neurons of brain [50]. Each path transmits a signal among neurons in a manner similar to that of synapses [51]. MLP, as a feedforward ANN, contains three main parts: one input layer, one or more hidden layers and one output layer, which can be successfully employed for prediction, classification, signal processing and error filtering [52]. Each node employs one nonlinear function. MLP employs backpropagation learning algorithm for training process [53,54]. MLP as popular and frequently used techniques among other MLPs was employed to predict the direction value. MLP was developed by the use of MATLAB software. Figure 1 indicates the architecture of developed network. Initially, the network divided data into two sets of training data (with a share of 80%) and testing data (with a share of 20%) randomly. In the first step of the training process, training to find the optimum number of neurons in the hidden layer. In each training process, Mean Square Error (MSE) was computed as the performance function.

Genetic algorithm (GA) and particle swarm optimization (PSO), as evolutionary algorithms, have been employed to train the neural network. This approach of hybridization of ANN has a lot of advantages such as increasing the accuracy of ANN by updating the weights and bias values using GA and PSO [55,56]. The aim of this study is to estimate the weights of hidden and output layers of an ANN architecture using GA and PSO during a convergence and accurate estimation process to generate accurate results, and, on the other hand, to control the deviation from target point in such a way that it prevents deviation and large errors even in different performances. However, the neural network needs to be left alone due to the random selection of a sample in order to arrive at an answer with appropriate accuracy. Therefore, this can be attributed to the stability and reliability of the neural network through GA and PSO.

#### 3.2.2. Genetic Algorithm (GA)

GA is a subset of approximation techniques in computer science to estimate a proper solution for optimization problems. GA is a type of evolutionary algorithm (EA) that employs biological techniques such as heredity and mutations [57,58]. The hypothesis begins with a completely random population and continues for generations. In each generation, the total population capacity is assessed, several individuals are selected in a random process from the current generation (based on competencies) and modified (deducted or re-combined) to form a new generation, and the next repetition of the algorithm becomes the current generation [59,60,61].

**Figure 2:**
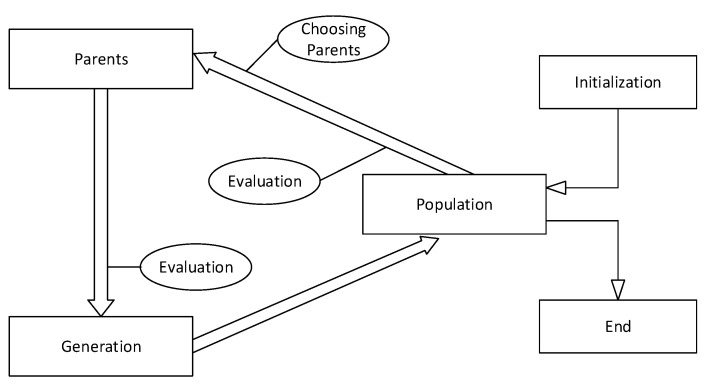
The implementation process of GA.

The optimization process in the genetic algorithm is based on a randomly guided process. This method is based on Darwin’s theory of gradual evolution and fundamental ideas. In this method, a set of objective parameters is randomly generated for a fixed number of so-called populations. Or the fit of that set of information is attributed to that member of that population [62,63,64]. This process is repeated for each of the created members, then formed by calling the operators of the genetic algorithm, including mutation and next-generation selection, and this process will continue until the convergence criterion is met [59,65]. There are three common criteria for stopping: Algorithm execution time, the number of generations that are created and the convergence of the error criterion. The process of implementing GA, which is the basis of evolutionary algorithms, is presented in Figure 2. which is adapted and regenerated from [66]. 

The main components of the Genetic algorithm include: representation of the environment, evaluation function, Population (set of answers), the process of choosing parents, Operators of Diversity (Generation), The process of selecting the living (choosing the best population to build the next generation) and stop condition. Genetic organization determines how each person displays themselves and behaves, and their physical quality. Differences in genetic organization are one of the criteria for distinguishing between different methods of evolutionary computation. The genetic algorithm uses linear binary organization. The most standard type of this organization is the use of an array of bits. Of course, an array of other types of data can also be used. This is due to their constant size. This facilitates integration operations [61,67,68]. However, it is possible to use variable length structures in organizing GA, which makes the implementation of integration very complex.

**Figure 3:**
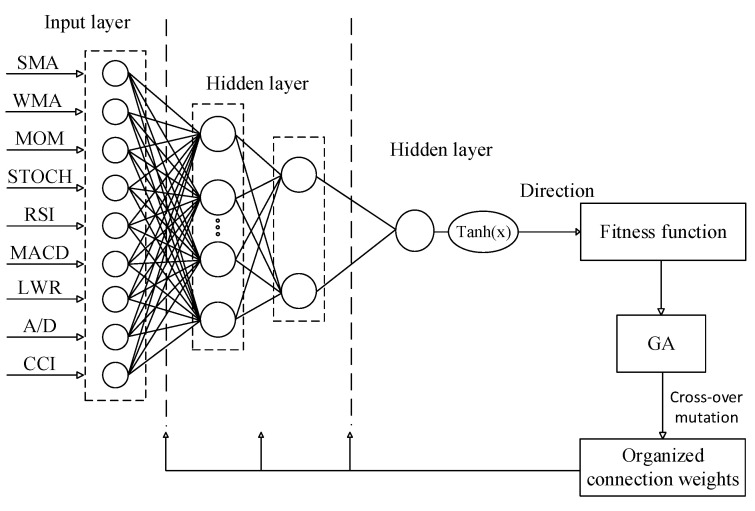
The flowchart of the MLP–GA algorithm.

In this research, the genetic algorithm was utilized to find the optimal point of complex nonlinear functions in integrating with the artificial neural network. Genetic algorithms optimize artificial neural network weights and bias values. In fact, the objective function of the genetic algorithm is a function of the statistical results of the MLP. To train, the P number of population of each generation, MLP was randomly initialized and the error rate was calculated using training data. In the next step, the network characteristics were updated according to the input and output values. The training process of the algorithm was repeated until the network features improved, taking into account the newly population. In the last step, the output gathered from the network execution was compared with the actual values and the model execution finished by minimizing the difference between the two values. Figure 3 presents the flowchart of the MLP–GA algorithm. Table 5 presents the setting parameters for GA.

#### 3.2.3. Particle Swarm Optimization (PSO)

PSO is a popular and robust optimization method to deal with problems in the *n*-dimensional space. The PSO is a mass search algorithm that is modeled on the social behavior of bird groups. Initially, the algorithm was employed for pattern detection of flight of birds at the same time and to suddenly change their path and optimize the shape of the handle. In PSO, particles flow in the search space which is affected by their experience and knowledge of their neighbors; thus the position of another particle mass affects how a particle is searched. Results of recognition of this behavior is the searching for particles to reach successful areas. The particles follow each other and move towards their best neighbors. In PSO particles are regulated throughout their neighborhood [69,70,71,72].

**Figure 4:**
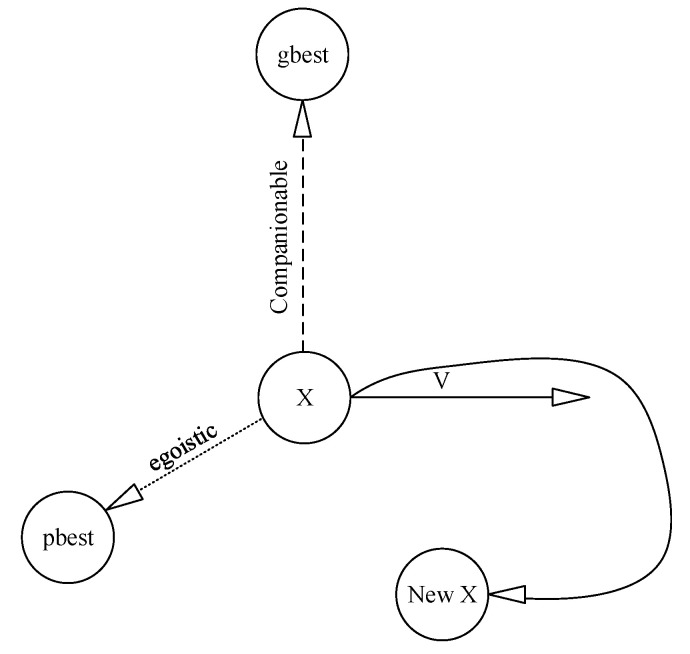
The performance of PSO.

At the beginning of the work, a group of particles are produced to reach the best solution. Each particle is updated through the finest position (pbest) and the finest position ever obtained by the particle population used by the algorithm (gbest) in each step, which is presented in Figure 4 based on an adaptation from [73,74,75,76,77]. Updating the velocity and location of each particle is the next step after finding the best values, Equations (1) and (2):

(1) v(t+1)=v(t)+c1×rand(t)×(pbest(t)−position(t)+c2×rand(t)×(gbest(t)−position(t))

(2) position(t+1)=v(t+1)+position(t)

Equation (1) has three parts in the right side, current particle velocity  v(t), the second part c1×rand(t)×(pbest(t)−position(t)) and third part c2×rand(t)×(gbest(t)−position(t)) are responsible for the rate of change of particle velocity and its direction towards the best personal experience (nostalgia) and the finest experience of the group (collective intelligence). If the first part is not considered in this equation v(t), then the velocity of the particles is determined according to the current position and the best particle experience, and in practice the effect of the current velocity and its inertia is eliminated. Accordingly, the best particle in the group stays in place, and the others move toward that particle. In fact, the mass movement of particles without the first part of Equation (1) will be a process in which the search space gradually shrinks and local search is formed around the best particle. The parameters *c*1 and *c*2 (the value is about 2) determine the importance and weight of collective intelligence and nostalgia [74,75,76]. As for the condition of stopping, the following ways are available:

- A certain number of repetitions,
- Achieve a decent threshold,
- A number of repetitions that do not change the competence (for example, if after 10 repetitions the competency was constant and did not improve),
- The last way is based on the aggregation density around the optimal point.

One of the advantages of PSO over GA is the simplicity and its low parameters. Selecting the best values for the cognitive and social component leads to accelerating the algorithm and preventing premature convergence occurs locally at optimal points. In PSO optimization, the proposed variables are included in the training of a neural network, including network weights and bias. The process is as follows: First, N vector of position Xi, which N is equal to the number of members of the category, is generated randomly. The neural network is executed according to the parameters equal to the variables of these vectors and the error obtained from each run is considered as the degree of fit of the variable vector of that network. This process is repeated till the final convergence is achieved. The ultimate convergence is to achieve the optimal position vector (values of optimal weights and bias) so that the training error is minimized. So the objective function in this optimization need to be minimized as the amount of error Forecast [78,79,80]. Table 6 presents the setting values of the MLP–PSO.

#### 3.2.4. Training Phase

Training process categorized into two main steps. First is to select the best architecture of the ANN, the second is to integrate MLP with optimizers. Therefore, training was developed with 10 to 19 neurons in the first hidden layer and 2 neurons in the second hidden layer with the 80 percent of total data, according to Table 7. MLP models called as Models 1–6. After this process, MLP was integrated with GA using population size 50, 100 and 150 (models 7–9, respectively) and PSO using particle size 50, 75, 100 and 125 (models 10–13, respectively). Training was performed into two scenarios, with Tanh (x) as the output function and with Gaussian function as the default function.

**Figure 5:**
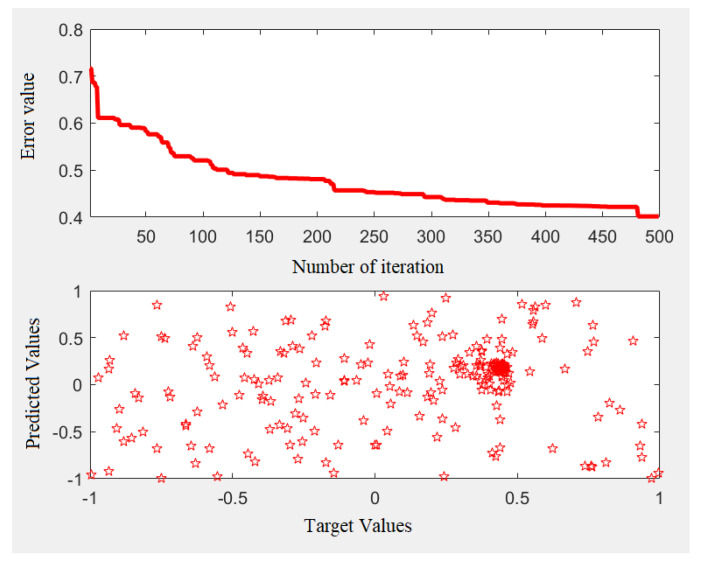
MLP–GA training process.

Figure 5 indicates a sample of the training process with MLP–GA which is extracted from the training process. The rest of data (20 percent) was employed in testing process.

#### 3.2.5. Evaluation Metrics

Table 8 presents evaluation criteria which compares predicted and output values. These equations are metrics for indicating the performance of models in predicting the target values as the model output values. In fact, this metrics compares the output of models and target values to calculate a value for indicating the accuracy of models [51,56,81].

## 4. Results

Training was performed by 80% of total data. Results were evaluated in terms of correlation coefficient, MAPE and RMSE, according to Table 9 and Table 10. Table 9 presents results of the training step without the use of Tanh (x) (using the Gaussian function as default) as the output function and Table 10 gives results of the training step with Tanh (x) as the output function.

As is clear from Table 9 and Table 10, Model 13 provides higher accuracy compared with other models. It is also clear that using Tanh (x) as the output function of MLP increases the accuracy of the prediction. According to the results, the hybrid methods increase the processing time (s) compared with those for the single methods. This was also claimed by Mosavi et al., 2019 [82] and Ardabili et al., 2019 [83]. The main reason can be due to the optimizing process on setting the weights and bias values of the MLP which consumes more processing time (s). On the other hand, according to the Table 9 and Table 10, it is clear that the GA requires more processing time compared with that of the PSO. More processing time also can be due to the complexity of the optimizers [84,85]. Using Tanh (x) reduced the processing time.

### Testing Results

Table 11 and Table 12 present the testing results, respectively, for the Gaussian function and with Tanh (x). as is clear, in testing process, results are different from those of training process. According to results of testing process, Model 7 followed by Model 13 for both scenarios, with Tanh (x) and Gaussian function, provide higher accuracy and lower error compared with other models. It is clear that the presence of Tanh (x) as the output function increases the accuracy and reduces the error values.

**Figure 6:**
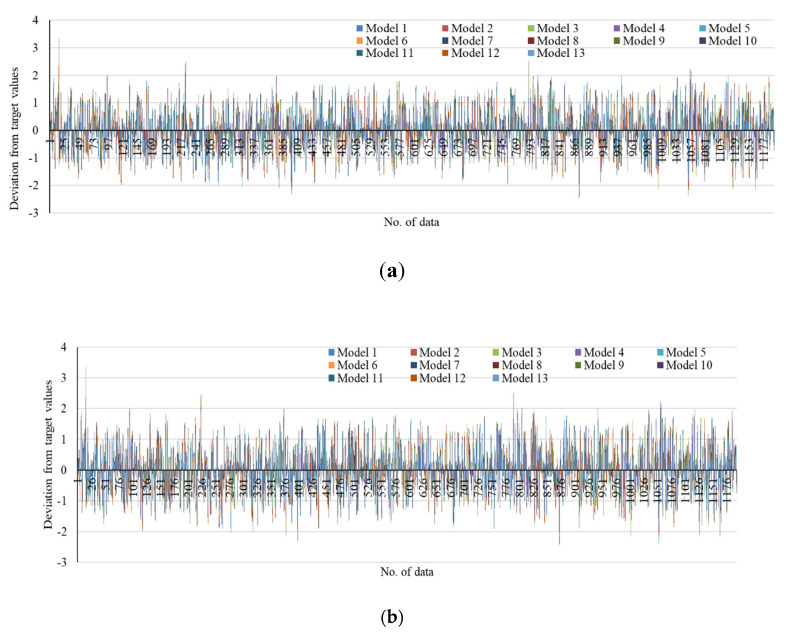

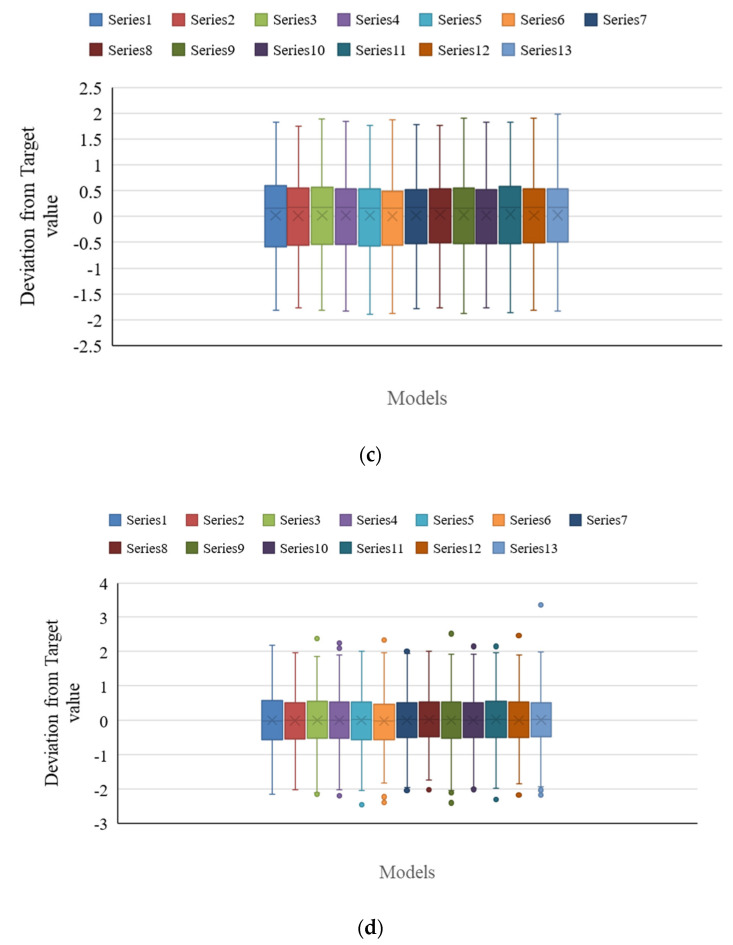
Deviation from target values for the developed models. (**a**,**d**) with the Gaussian function as default, (**b**,**c**) with the Tanh (x) function.

Figure 6 presents the deviation from target values of all models into two scenarios with the Gaussian function as the default and with Tanh(x) as the output function. According to Figure 6, it can be concluded that the presence of Tanh (x) as the output function reduces the range of deviation. Models with a high accuracy have lower deviation values compared to others. 

**Figure 7:**
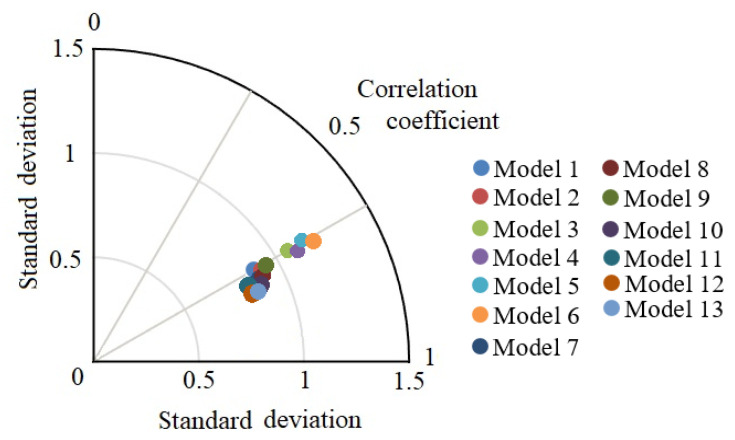
Taylor diagram for the best response of the models in testing step.

Figure 7 indicates a simple yet essential form of Taylor diagram for the testing process of the developed models. This diagram is developed according to correlation coefficient and standard deviation. A point with a lower standard deviation and higher correlation coefficient has higher accuracy compared with other points. As is clear from Figure 7, Model 13 and Model 7 present higher accuracy compared with other models.

According to the results, the advantage of the single models is their lower processing time, but the lowest accuracy can be the most important limitation and disadvantage of the single models compared with the hybrid ones. This was also claimed by several researches. In the case of using the hybrid models, the advantages of MLP–PSO such as higher accuracy and lower processing time overtake the MLP–GA.

## 5. Conclusions

In this paper, modeling was performed by MLP–GA and MLP–PSO in two scenarios including with Tanh (x) and with the Gaussian function as default as the output function in thirteen categories. Research outcomes were evaluated using RMSE and correlation coefficient values to compare the accuracy and performance of the developed models in training and testing steps. Based on the results, using Tanh (x) as the output function improved the accuracy of models significantly. MLP–PSO with population size 125 followed by MLP–GA with population size 50 provided higher accuracy in the testing step by RMSE 0.732583 and 0.733063, MAPE of 28.16%, 29.09% and correlation coefficient 0.694 and 0.695, respectively. As is clear, the only advantage of the single MLP is its lower processing time but the important disadvantage can be claimed the lower accuracy compared with the hybrid models. According to the results, using hybrid ML method could successfully improve the prediction accuracy. Accordingly, MLP–PSO with lower processing time and higher accuracy (as the main advantage of the PSO compared with GA) overtakes the MLP–GA. In this way, the problem statements were successfully covered by the solution presented in the study. The main limitation for the future, is about presenting and beating a new stock market index using evolutionary methods. The future work will address the beating of stock market, that the variance of stock market will be successfully addressed. Thus, the return variance poses a limitation of the present research.

## Figures and Tables

**Table 1:** Stock market index direction forecasting with machine learning considering comparative analysis involving ANN-based methods.

References	Method/s	Application/Data	Result
[15]	ANN, ARIMA	KLCI (1984–1991)	ANN outperformed ARIMA model.
[16]	SVM, BPNN	KOSPI (1989–1998)	SVM outperformed ANN.
[32]	ICA–BPNN, BPNN	TAIEX (2003–2006)	ICA–BPNN is superior.
[17]	ANN, NB, DT	BSE (2003–2010)	Hybrid RSs outperformed ANN.
[34]	PNN, SVM	S&P 500 (2000–2008)	PNN provided high accuracy.
[24]	ANN, SVM	BIST 100 (1997–2007)	75% accuracy using ANN.
[29]	ANN	TEPIX (2002–2009)	ANN showed promising results.
[35]	ANN, GA	TEPIX (2000–2008)	ANN delivered next day estimates.
[25]	ANN	BIST 100 (2002–2007)	ANN achieved success with 82.7%.
[36]	SVM, ANN	IBEX-35 (1990–2010)	SVM outperformed ANN.
[37]	*k*-NN, PNN	S&P 500 (2003–2008)	*k*-NN outperformed PNN.
[18]	ANN	BOVESPA (2000–2011)	ANN suitable for direction estimation.
[38]	LSSVM, PNN,	CSI 300 (2005–2012)	LSSVM outperformed other models.
[39]	Random walk, ANN, SVM, fuzzy	BSE-SENSEX (2011–2012)	The fuzzy metagraph-based model has reached a classification rate of 75%.
[40]	ANN, RF, *k*-NN	Amadeus (2009–2010)	RF outperformed ANN.
[20]	NB, ANN, SVM	CNX Nifty (2003–2012)	NB outperformed other models.
[41]	DWT, ANN, SVM–MLP	DJIA-S&P500 (2000–2012)	SVM–MLP is superior.
[42]	Probit, Logit, Extreme Value	S&P 500 (2011–2015)	Extreme Value outperfomed Logit and Probit.
[7]	PSO–ANN	S&P 500, IXIC (2008–2010)	Acceptable prediction and robustness.
[43]	RF & ANN	S&P 500 (2009–2017)	RF outperformed ANN.
[44]	Hybrid fuzzy NN	DAX-30 (1999–2017)	minimum risky strategies.
[45]	GA, SVM, ANN	BM&FBOVESPA PETR4 (1999–2017)	SVM performed better than ANN.

**Table 2:** Stock market index direction studies using methods other than ANNs.

Author/s	Method/s	Application	Result
[31]	GA–SVM, random walk, SVM, ARIMA, BPNN	S&P 500 (2000–2004)	GA–SVM has been shown to outperform other models.
[14]	Fuzzy sets, physical, support vector regression, partial least squares regression	TAIEX and HIS (1998–2006)	Their proposed models outperform the compared models according to the RMSE.
[46]	Random forest	CROBEX (2008–2013)	Random forests can be successfully preferred to estimate.
[19]	Fuzzy rule-based expert system	Apple company (2010–2014)	The fuzzy expert system has significant performance with minimal error.
[21]	GMM–SVM	Indonesia ASII.JK (2000–2017)	The GMM–SVM model has been found to be superior to other models.
[47]	Bayesian network	iBOVESPA (2005–2012)	Mean accuracy with the proposed model configuration was almost 71%.
[48]	TOPSIS, SVM, NB, Decision tree, kNN	BSE SENSEX, S&P500 (2015–2017)	While SVM model performs better in BSE SENSEX index, *k*-NN is superior to other models in S&P 500 index.
[22]	ANFIS	Apple stock data (2005–2015)	The proposed method outperformed the existing methods.
[49]	RKELM	BSE, HIS, FTSE (2010–2015)	They proved the superiority of the RKELM model over the ANN, naive Bayes and SVM.
[3]	Mean Profit Rate (MPR)	DJIA, S&P500, HSI, Nikkei 225, SSE (2007–2017)	MPR is an effective classifier.

**Table 3:** Technical indicators used in SM direction estimation.

Author/s	Technical Indicators
[15]	Simple moving average (SMA), stochastic K (%K), momentum (MOM), stochastic D (%D), relative strength index (RSI).
[16]	Slow D%, MOM, rate of change (ROC), K%, Larry William’s R% (%R), Accumulation/Distribution (A/D) oscillator, disparity5, RSI, disparity10, price oscillator (OSCP), D%, Commodity Channel Index (CCI).
[31]	OSCP, Stochastic oscillator (SO), Slow stochastic oscillator (SSO), CCI, ROC, MOM, SMA, Moving variance (MV), Moving variance ratio (MVR), Exponential moving average (EMA), Moving average convergence and divergence (MACD), A/D oscillator, Price (P), disparity5, disparity10, Moving stochastic oscillator (MSO), RSI, linear regression line (LRL).
[32]	The previous day’s cash market high, low, volume, 6-day RSI, today’s opening cash index, 10-day total amount weighted stock price index.
[17]	%K, Positive volume index, %R, negative volume index, %D, on balance volume, RSI, MACD, MOM, A/D oscillator, 25-day SMA.
[34]	SMA, OSCP, MOM, %D, ROC, disparity, %K.
[29]	MACD, SMA, %R, CCI, A/D oscillator, %D, weighted moving average (WMA), RSI, MOM, %K.
[24]	%D, %K, RSI, MOM, MACD, WMA, %R, A/D oscillator, SMA, CCI.
[35]	SMA, MACD, RSI, OSCP, MOM, volume.
[18]	MACD, RSI, %D, SMA, Bollinger band, MOM, %R.
[14]	SMA for 5 days, SMA for 10 days, bias to moving average (BIAS), RSI, psychological line (PSY), %R, MACD, MOM.
[38]	%K, %R, %D, CCI, A/D oscillator, MOM, MACD, RSI, SMA and WMA.
[39]	MA, exponential moving average (EMA), MACD, RSI.
[19]	High price, low price, volume, change of closed price, MACD, MA, BIAS, RSI, %R.
[20]	%D, RSI, WMA, MACD, CCI, A/D oscillator, %K, %R, SMA.
[46]	5-day SMA, 5-day WMA, 10-day SMA, 10-day WMA, %K, %D, MACD, CCI, 5-day disparity, 10-day disparity, OSCP, ROC, MOM, RSI, 5-day standard deviation.
[41]	SMA, EMA, A/D oscillator, %K, RSI, OSCP, closing price, maximum price.
[42]	SMA, WMA, MOM, %K, %D, %R, RSI, MACD.
[7]	Change of price, change of volume, 5-day SMA, 10-day SMA, 30-day SMA, moving price level (30 days), moving price level (120 days), percentage price oscillator.
[21]	A/D oscillator, mean of rising days, CCI, SMA, MACD, MOM, on balance volume, ratio of rising days, RSI, %R.
[44]	Triangular moving average (TMA), RSI, SMA, EMA, modified moving averages (MMA), volatility ratio (VR), %R, true strength index (TSI), average true range (ATR).
[48]	SMA, %K, %D, %R, MACD, RSI.
[45]	SMA, WMA, MOM, RSI.
[22]	1-week SMA, 2-week SMA, 14-day disparity, R%.
[3]	%D, %K, RSI, MOM, MACD, WMA, %R, A/D oscillator, SMA, CCI.
[49]	SMA, MACD, %K, %D, RSI, %R.

**Table 4:** Selected technical indicators.

Technical Indicators	Abbreviation	Formulas
Simple *n* (10 here)-day Moving Average	SMA	$SMA=\frac{C_{t}\;+\;C_{t\;-\;1}+\cdots +\;C_{t\;-\;n}}{n}$
Simple *n* (10 here)-day Moving Average	WMA	$WMA=\frac{10\;\times \;C_{t}\;+\;9\;\times \;C_{t-1}+\cdots +C_{1}}{n\;+\;\left(n\;-\;1\right)\;+\cdots +\;1}$
Momentum	MOM	$MOM=C_{t}-C_{t-9}$
Stochastic D%	STOCH	$Stokastik\%D=\frac{\sum_{i=0}^{n-1}K_{t-i}}{10}\%$
Relative Strength Index	RSI	$RSI=100-\frac{100}{1+(\sum_{i=0}^{n-1}UP_{t-i}/n)/(\sum_{i=0}^{n-1}DW_{t-i}/n)}$
Moving Average Convergence Divergence	MACD	$MACD=MACD{\left(n\right)}_{t-1}+\frac{2}{n\;+\;1}\times (DIFF_{t}-MACD\left(n{)}_{t\;-\;1}\right)$
Larry William’s R%	LWR	$William\prime s\%R=\frac{H_{n}\;-\;C_{t}}{H_{n}\;-\;L_{n}}\times 100$
Accumulation/Distribution Oscillator	A/D	$A/D=\frac{H_{t}\;-\;C_{t\;-\;1}}{H_{t}\;-\;L_{t}}$
Commodity Channel Index	CCI	$CCI=\frac{M_{t}\;-\;SM_{t}}{0.015D_{t}}$

*C_t_* is the closing price, *L_t_* the low price, *H_t_* the high price at time *t*, DIFF: EMA(12)_t_– EMA(26)_t_, EMA exponential moving average, $EMA=a\times x_{t}+\left(1-a\right)\times x_{t-m}$, α smoothing factor: 2/(1 + *k*), *k* is time period of *k* day exponential moving average, *LL_t_* and *HH_t_* mean lowest low and highest high in the last *t* days, respectively, *M_t_*: (*H_t_*+*L_t_*+*C_t_*)/3; $SM_{t}:\left(\sum_{i=1}^{n}M_{t-i+1}/n\right)$, $D_{t}=\left(\sum_{i=1}^{n}\left|M_{t-i+1}-SM_{t}\right|\right)/n$, *Up_t_* means the upward price change, *Dw_t_* means the downward price change at time *t*.

**Table 5:** The characteristics of the MLP–GA.

Input Neuron	9
Hidden layer	2
Hidden layer activation function	Logsig
Output layer activation function	Gaussian, Tanh (x)
Pop. type	Double vector
Pop. size	50, 100 and 150
Crossover function	Scattered
Crossover fraction	0.8
Selection function	Uniform
Migration interval	10
Migration fraction	0.2

**Table 6:** The characteristics of the MLP–PSO.

Input Neuron	9
Hidden layer	2
Hidden layer activation function	logsig
Output layer activation function	Gaussian, Tanh (x)
Number of Max. Iteration	500
Pop. size	50, 75, 100 and 125
c_1_	2
c_2_	2

**Table 7:** The description of the developed models.

Model 1	MLP (9-10-2-1)	Model 8	MLP–GA (100)
Model 2	MLP (9-12-2-1)	Model 9	MLP–GA (150)
Model 3	MLP (9-14-2-1)	Model 10	MLP–PSO (50)
Model 4	MLP (9-15-2-1)	Model 11	MLP–PSO (75)
Model 5	MLP (9-17-2-1)	Model 12	MLP–PSO (100)
Model 6	MLP (9-19-2-1)	Model 13	MLP–PSO (125)
Model 7	MLP–GA (50)		

**Table 8:** Model Evaluation metrics.

Accuracy and Performance Index	Description
Correlation coefficient = $\frac{N\sum_{\;}^{\;}{\left(XY\right)}^{\;}-\sum_{\;}^{\;}{\left(X\right)}^{\;}\sum_{\;}^{\;}{\left(Y\right)}^{\;}}{\sqrt{[N\sum_{\;}^{\;}X^{2}-{\left(\sum_{\;}^{\;}X\right)}^{\;}{}^{2}]{\left[N\sum_{\;}^{\;}X^{2}-{\left(\sum_{\;}^{\;}XY\right)}^{\;}{}^{2}\right]}^{\;}{}^{\;}}}$	N: Number of Data X: Target value Y: Output value.
$\mathrm{MAPE}\;\left(\%\right)=\frac{1}{N}\overset{N}{\underset{i=1}{\sum }}\left|\frac{X_{i}-Y_{i}}{X_{i}}\right|$	
RMSE = $\sqrt{\frac{1}{N}\sum_{\;}^{\;}{\left(X-Y\right)}^{2}}$	

**Table 9:** Results for training phase with the Gaussian function as default.

Model	Correlation Coefficient	RMSE	MAPE (%)	Processing Time (s)	Model	Correlation Coefficient	RMSE	MAPE (%)	Processing Time (s)
Model 1	0.67	0.741035	32.02%	3.82	Model 8	0.694	0.718928	30.57%	8.33
Model 2	0.68	0.733079	31.55%	4.11	Model 9	0.70	0.713458	30.31%	10.82
Model 3	0.676	0.735209	31.52%	4.97	Model 10	0.692	0.721568	30.40%	6.78
Model 4	0.682	0.730448	30.88%	5.11	Model 11	0.689	0.724479	30.89%	7.32
Model 5	0.689	0.723326	30.84%	5.22	Model 12	0.693	0.720478	30.28%	9.02
Model 6	0.693	0.719818	30.59%	5.30	**Model 13**	**0.704**	**0.708774**	**29.93%**	**10.03**
Model 7	0.692	0.720763	30.59%	7.22					

**Table 10:** Results for training phase with tanh(x) function.

Model	Correlation Coefficient	RMSE	MAPE (%)	Processing Time (s)	Model	Correlation Coefficient	RMSE	MAPE (%)	Processing Time (s)
Model 1	0.684	0.745674	30.12%	3.82	Model 8	0.709	0.72298	28.79%	7.96
Model 2	0.692	0.738467	29.59%	4.11	Model 9	0.716	0.717001	28.69%	9.87
Model 3	0.69	0.739543	29.62%	4.97	Model 10	0.710	0.720822	28.93%	6.22
Model 4	0.698	0.730832	29.09%	5.11	Model 11	0.703	0.728695	29.03%	7.12
Model 5	0.709	0.724664	29.23%	5.22	Model 12	0.708	0.721266	28.48%	8.45
Model 6	0.707	0.724155	28.66%	5.30	**Model 13**	**0.720**	**0.712372**	**28.16%**	**9.23**
Model 7	0.708	0.723435	28.78%	7.05					

**Table 11:** Results for testing phase with the Gaussian function as default.

Model	Correlation Coefficient	MAPE (%)	RMSE		Correlation Coefficient	MAPE (%)	RMSE
Model 1	0.648	32.63%	0.759687	Model8	0.681	31.00%	0.730846
Model 2	0.661	32.11%	0.748273	Model 9	0.664	31.44%	0.746575
Model 3	0.657	32.07%	0.752376	Model 10	0.680	31.11%	0.731245
Model 4	0.673	31.31%	0.737857	Model 11	0.663	31.89%	0.747604
Model 5	0.663	31.71%	0.747776	Model 12	0.678	30.97%	0.733873
Model 6	0.671	31.24%	0.740842	Model 13	0.677	31.04%	0.735221
**Model 7**	**0.681**	**30.84%**	**0.729959**				

**Table 12:** Results for testing phase with Tanh (x).

Model	Correlation Coefficient	MAPE (%)	RMSE	Model	Correlation Coefficient	MAPE (%)	RMSE
Model 1	0.662	30.92%	0.76042	Model 8	0.692	29.10%	0.734701
Model 2	0.673	30.15%	0.751537	Model 9	0.679	29.95%	0.744885
Model 3	0.669	30.13%	0.753864	Model 10	0.694	29.50%	0.73393
Model 4	0.688	29.54%	0.738487	Model 11	0.674	30.10%	0.749981
Model 5	0.670	30.29%	0.74539	Model 12	0.694	29.20%	0.733235
Model 6	0.684	29.48%	0.740869	**Model 13**	**0.694**	**29.09%**	**0.732583**
Model 7	0.695	29.16%	0.733063				

## Abbreviations

MLP	Multilayer perceptron	TEPIX	Tehran stock exchange price index
MV	Moving variance	LDA	linear discriminant analysis
GA	Genetic algorithms	BPNN	Back propagation neural network
RMSE	Root mean square error	BSE-SENSEX	Bombay Stock Exchange
ANN	Artificial neural network	TAPI 10	10-day total amount weight stock price index
MOM	momentum	ICA	Independent Component Analysis
SVM	Support vector machine	TAIEX	Taiwan Stock Exchange Capitalization Weighted Stock Index
ROC	rate of change	KOSPI	Korea Composite Stock Price Index
BIST 100	Borsa Istanbul 100 index	MAPE	Mean Absolute Percentage Error
%D	stochastic D	CSI 300	Capitalization-weighted SM index
CROBEX	Zagreb stock index	CNX Nifty	Standard & Poor’s CNX Nifty stock index
DAX-30	German DAX-30	DJIA	Dow Jones Industrial Average
HIS	Hang Seng Index	FTSE	Financial Times Stock Exchange
*k*-NN	*k*-nearest neighbor	QDA	quadratic discriminant analysis
S&P 500	Standard & Poor’s 500	GMM	Gaussian mixture model
RBF	Radial basis function	RKELM	Robust kernel extreme learning machine
SMA	simple moving average	KLCI	Kuala Lumpur Composite Index
RSI	relative strength index	BOVESPA	Bolsa de Valores de São Paulo
%K	stochastic K	PNN	Probabilistic neural network
%R	Larry William’s R%	A/D	Accumulation/Distribution
OSCP	price oscillator	CCI	Commodity Channel Index
SO	Stochastic oscillator	MSO	Moving stochastic oscillator
SSO	Slow stochastic oscillator	PSO	Particle swarm optimization
MVR	Moving variance ratio	EMA	Exponential moving average
LRL	Linear regression line	MACD	Moving average convergence and divergence
NB	Naive Bayes	SM	Stock market
RS	Rough sets	DWT	Discrete wavelet transform
IBEX-35	Spanish SM	ARIMA	Autoregressive integrated moving average
